# Qualitative Metabolite Profiling of *Orchis purpurea* Huds. by GC and UHPLC/MS Approaches

**DOI:** 10.3390/plants13081064

**Published:** 2024-04-10

**Authors:** Valeria Cavalloro, Stefania Pagliari, Fabio Gosetti, Luca Campone, Cristina Sottani, Simona Collina, Emanuela Martino, Francesco Saverio Robustelli della Cuna

**Affiliations:** 1 Department of Earth and Environmental Sciences, University of Pavia, Via Ferrata 1, 27100 Pavia, Italy; valeria.cavalloro@unipv.it; 2 NBFC—National Biodiversity Future Center, Piazza Marina 61, 90133 Palermo, Italy; fabio.gosetti@unimib.it (F.G.); luca.campone@unimib.it (L.C.); 3 Department of Biotechnology and Biosciences, University of Milano-Bicocca, Piazza Della Scienza 2, 20126 Milan, Italy; s.pagliari2@campus.unimib.it; 4 Department of Earth and Environmental Sciences, University of Milano-Bicocca, Piazza Della Scienza 1, 20126 Milan, Italy; 5 Environmental Research Center, ICS Maugeri SPA SB, Institute of Pavia, IRCCS, Via Maugeri 2, 27100 Pavia, Italy; cristina.sottani@icsmaugeri.it (C.S.); fsaveriorobustelli@unipv.it (F.S.R.d.C.); 6 Department of Drug Sciences, University of Pavia, Viale Taramelli 12, 27100 Pavia, Italy; simona.collina@unipv.it

**Keywords:** *Orchis purpurea*, secondary metabolites, essential oil, coumarin, UHPLC-MS/MS

## Abstract

Orchids are experiencing wide success in ornamental, medicinal, and food fields. The reason for their success is correlated with both their morphology and metabolomics, the latter linked to their taste and biological effects. Despite many orchids having already been the subject of chemotaxonomic works, some of them are still untapped, like the case of *Orchis purpurea*. *O. purpurea* is one of the most common species of the genus *Orchis*, present in hedgerows, verges, and light woodland, where it is one of the few herbaceous plants able to be unpleasant to herbivorous animals. Essential oil from roots, stems, leaves, and flowers were analyzed via GC/MS analyses, revealing the presence of 70 compounds, with a clear prevalence of coumarin. The high concentration of this metabolite may explain the resistance of *O. purpurea* to herbivores, being associated with appetite-suppressing properties and a bitter taste. Non-volatile fractions were analyzed via UHPLC-MS analysis revealing the presence of hydroxycinnamic acid derivatives, polyphenols, and glycosidic compounds, probably responsible for their color and fragrance. Taken together, the herein presented results shed light on both the defensive strategy and the chemotaxonomy of *O. purpurea*.

## 1. Introduction

Orchids are well-known ornamental plants, appreciated worldwide, and ranked among the best sellers in the global potted plant trade. Their beautiful flower is the reason for their commercial success, even if orchids are also well-known for other applications [1,2]. Particularly, orchids are the ingredient of traditional products such as Chikanda, Faham, and Salep [1,3]. This latter is a powder obtained from the dried tubers of more than 35 species of terrestrial orchids, including species from the genera *Anacamptis*, *Dactylorhiza*, and *Orchis*, to cite just a few [4]. Its high consumption in the eastern Mediterranean area is causing serious risks to both consumers and orchids [5]. Thus, tubers are often illegally harvested causing conservation concerns [6], while the final product may be adulterated with potential health risks [7].

Among orchids, the genus *Orchis* has a high diversity with about 20 terrestrial species. They are characterized by two egg-shaped underground tubers and one spike with flowers of different colors, and most species have several narrow leaves at the base [8].

**Figure 1:**
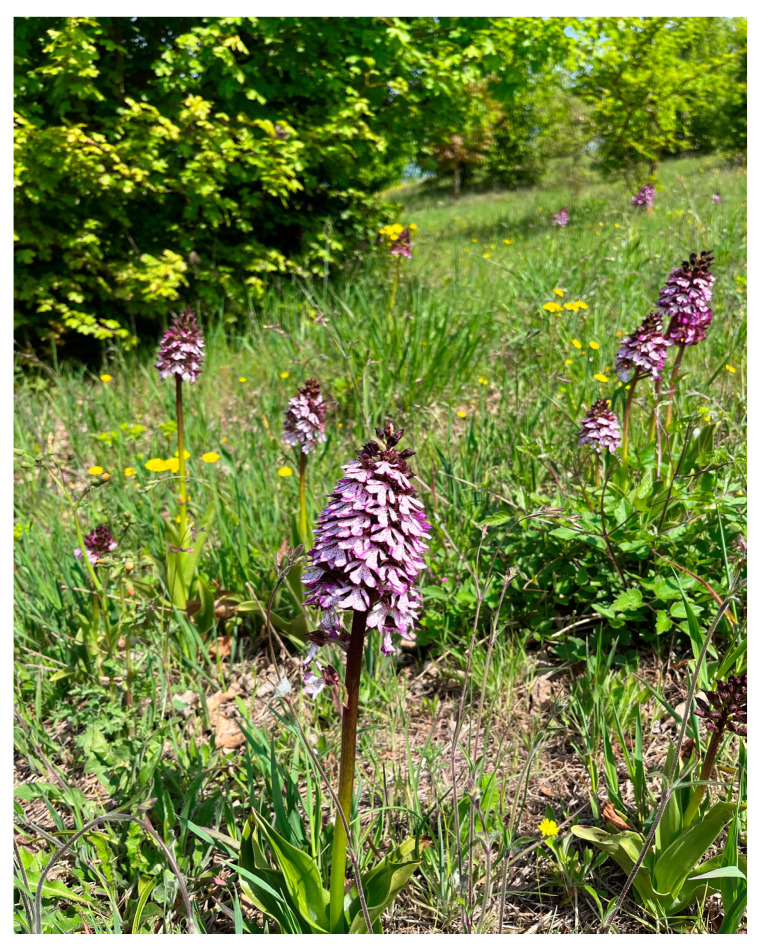
*Orchis purpurea* in its natural habitat in Pianlago Ponzone (Italy). Photo courtesy of Carlo Ugolotti.

One of the most common species of the genus *Orchis* is *Orchis purpurea* Huds (Asparagales: *Orchidaceae*). This species was first described in 1762 and it is widely spread in Central Asia, Europe, and North Africa. *O. purpurea* grows on alkaline soils and favors slightly shaded locations such as hedgerows, verges, and light woodland. Its name refers to the purple inflorescence, which is also the reason for its common name Lady Orchid. *O. purpurea* is one of the tallest and most robust European orchids, being able to reach almost one meter in height (Figure 1). This plant is present on almost all the Italian territory, with few exceptions (i.e., Valle d’Aosta and Sicily regions), and it can be mainly found in mature and luminous forests, and in semi-natural herbaceous vegetation. Interestingly, in these environments, *O. purpurea* is one of the few herbaceous plants able to resist herbivorous animals.

Despite its long-lasting presence in checklists of different territories and, more generally, the great interest of the scientific community in its belonging genus, *O. purpurea* remains underexplored. Thus, using “*Orchis purpurea*” as keywords on Scopus, only 51 articles were retrieved (update January 2024), mainly related to floristic checklists, morphological characterization, or behavior vs. climate change. Its first preliminary metabolomic characterization was published only in 2022, and it referred only to the volatile fraction obtained by the inflorescences [9]. Despite its preliminary nature, this work allowed us to highlight an interesting and peculiar trait of *O. purpurea*: the main compound identified in the inflorescences’ essential oils was coumarin. Coumarin is a well-known secondary metabolite that can be found in many different genera and species. Its main physiological role is the defense of the producing organism against both biotic and abiotic stress [10,11]. This metabolite is generally stored inside the vacuoles in its glycosylated form, while the aglycone is usually formed after stress.

To the best of our knowledge, coumarin has never been isolated from the genus *Orchis* thus far and it has been recently considered an underexplored metabolite in the Orchidaceae family [12]. Consistently, in this work, we wanted to deepen its presence in different organs of *O. purpurea* and to draw the first complete metabolomic fingerprint of the volatile fraction of this underestimated plant. Furthermore, a preliminary investigation of the non-volatile fraction has also been performed.

## 2. Results and Discussion

### 2.1. Essential Oil Characterization

After the unambiguous identification of previously collected *Orchis purpurea* [8], we extracted its main parts by steam distillation and obtain the volatile fraction via liquid/liquid extraction. The yields of *O. purpurea* essential oil from fresh roots, stems, leaves, and flowers were 0.009%, 0.03%, 0.09%, and 0.02% (weight of essential oils/weight of fresh material × 100), respectively. As can be noticed, leaves allowed us to obtain higher amounts of essential oils, followed by stems, flowers, and finally roots. GC/MS analyses revealed the presence of 70 compounds, listed in their elution order and reported as percentages of the total EO. The qualitative and quantitative results on the Elite-5MS column are reported in Table 1.

Coumarin was confirmed to be the most abundant secondary metabolite present in all the natural matrices except for roots, where the most abundant metabolite is (*E*)-15-heptadecenal (Table 1). Particularly, it represented almost the only metabolite present in the essential oil from leaves (98.86%), more than a half of the essential oil from stems and flowers (86.10% and 69.72%, respectively), and finally almost one-third of the essential oil from roots (32.30%). Simple coumarin (2H-1-benzopyran-2-one) and coumarin-derived compounds are widespread in the natural kingdom, especially in the Umbelliferae, Rutaceae, Oleaceae, Orchidaceae Moraceae, and Compositae families [13]. Still, it has also been detected in microorganisms, sponges, and animal species [14,15]. Focusing on orchids, these compounds are present in the essential oil derived from flowers and leaves of both epiphytic (i.e., *Dendrobium moschatum*, and *D. amabile*) and terrestrial orchids (i.e., *Anacamptis morio*, and *Ophrys sphegodes*) [9,16,17]. The wide diffusion of these metabolites in the natural kingdom can be easily explained considering their biological properties. Coumarins exhibit appetite-suppressing properties and a bitter taste able to protect the producing organism from herbivores [18]. Moreover, coumarins are also endowed with antimicrobial agents and can be released in the rhizosphere or accumulated in other organs after stress. All these activities related to coumarins may explain why *O. purpurea* is particularly able to resist herbivorous animals.

The other classes of compounds present in the different parts of *O. purpurea* are detailed below.

Roots: the essential oil was characterized by a high content of aldehydes (44.28%), dominated by (*E*)-15-heptadecenal (43.39%). The second largest class was represented by alcohols (16.12%), from which 1-hexadecanol (14.03%) and *p*-cresol (1.0%) were the most abundant compounds.

Stems: the major constituents of the essential oil were found to be alcohols (11.91%), from which *p*-cresol (11.58%) and 2-phenyl-2-propanol (0.11%) were the most representative compounds. Saturated hydrocarbons (1.04%) were represented by pentacosane (0.18%) and heptacosane (0.14%).

Leaves: the essential oil from leaves was dominated by coumarin (98.86%), followed by alcohols (0.61%), from which *p*-cresol (0.44%) was the most abundant compound.

Flowers: the most abundant class was represented by alcohols (15.13%) from which *p*-cresol (12.68%), *p*-vinylphenol (1.20%), and *p*-methylguaiacol (0.37%) were the most abundant compounds. The second largest class was represented by saturated hydrocarbons (7.13%).

**Figure 2:**
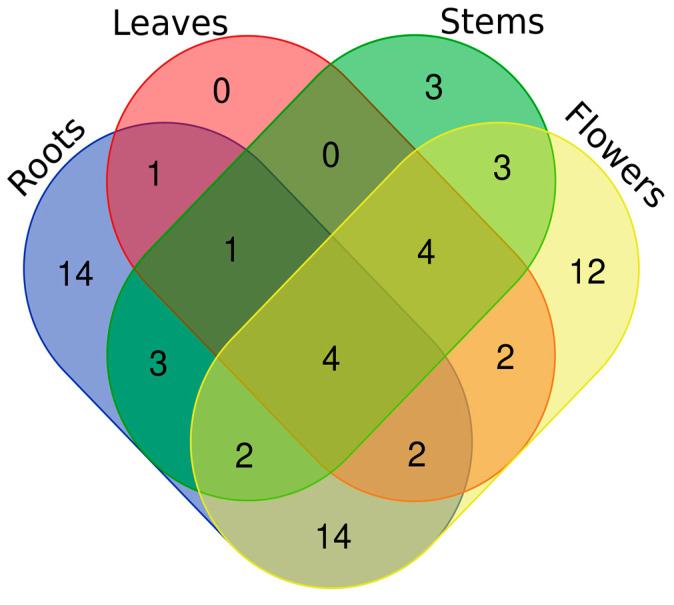
Venn diagram with the number of compounds shared between different parts of *O. purpurea*.

Except for coumarins, flowers resulted in the most diversified oils in terms of chemical composition (Table 2). On the other hand, roots were particularly rich in long chain acids, alcohol, and aldehydes, confirming their storage function. Of particular interest, *p*-cresol was produced in considerable amounts by both stems (11.94%) and flowers (12.88%). This secondary metabolite could represent a further defense for the plant, being considered a toxin with phytotoxic allelopathic activity [19]. Another valuable hypothesis suggest that *p*-cresol is produced due to its ability to specifically attract specific pollinators [20,21]. A Venn diagram (Figure 2) was realized to illustrate qualitative similarities and differences in volatile profiles among the different parts of *O. purpurea* [22].

As highlighted in Figure 2, a core of 12 compounds (18.4% of the total number of compounds detected) was present only in flowers, 3 (4.61%) in stems, and 14 (21.5%) in roots. In contrast, leaves do not show any specific compounds, but share some with flowers, roots, and stems.

### 2.2. Characterization of the Non-Volatile Constituents

Once we had characterized the volatile portion of *O. purpurea*, we focused our attention on the characterization of the non-volatile constituents. Thus, to the best of our knowledge, only the non-volatile fraction obtained by the hypogeal part of *O. purpurea* has already been analyzed in previous work [23]. Consistently, both leaves and flowers were sequentially extracted by exploiting a Soxhlet apparatus with *n*-hexane, ethyl acetate, and methanol. The three obtained fractions were evaporated under vacuum and the resulting yields are reported in the table hereunder (Table 3).

**Figure 3:**
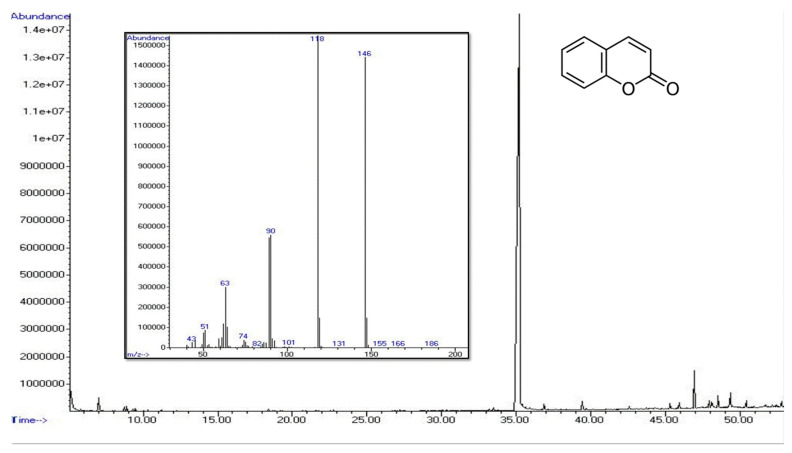
GC-MS chromatographic profile of *n*-hexane extract of leaves of *O. purpurea* and GC-MS EI spectrum of the main peak.

As expected, the three fractions contained completely different metabolites. Particularly, *n*-hexane fraction obtained from leaves and flowers, contained almost only coumarin, as demonstrated by GC-MS (Figure 3), while the ethyl acetate fraction allowed us to obtain a very low yield, and its analytical fingerprint was not significant [24,25].

Different results were obtained on the methanolic fraction. Thus, the UHPLC-MS/MS analysis highlighted that both leaves and flowers contain hydroxycinnamic acid derivatives (i.e., p-coumaroyl derivatives) and polyphenols (i.e., quercetin, luteolin, and kaempferol derivatives) as reported in Table 4. Furthermore, Dactylorhin A and Militarine, both glycosidic compounds, are compounds already found in other orchids belonging to the genus *Bletilla*, *Pleione*, and *Coeloglossum*.

Some secondary metabolites are characterizing of only the leaves or flowers.

Compound stored only in the flowers are Coelovirin E, a kaempferol glycoside, saponins, and Cyanidin-3-O-glucoside. Particularly, this last compound belongs to the class of anthocyanins, which are metabolites that cause a broad spectrum of orchid flower coloration.

On the other hand, Koaburaside and Coelovirin D are present only in the leaves. In detail, Coelovirins B, D, and E are tartrate derivatives already identified in another orchid belonging to genus *Coeloglossum*, while Koaburaside is a glycosilated phenolic compound already identified in other organism as *Fallopia multiflora* [26].

**Table 4:** Identified compounds by UHPLC-MS/MS. The retention time, precursor ion (negative or positive), compound name, chemical formula, MS error (ppm), characteristic product ions, and analyzed extract are reported.

N°	Rt (min)	[M − H]^−^	[M + H]^+^	Compounds	Formula	Mass Error (ppm)	Product Ions	Flowers/Leaves	Ref.
1	2.22	331.1033		Koaburaside	C_14_H_20_O_9_	−0.5	123.0445/105.0338	leaves	[27]
2	3.59	205.0855		Unknown	C_12_H_13_O_3_	−4.9	129.0554/115.0761	Flowers/leaves	-
3	4.22	367.1247		Coelovirins E	C_14_H_2_4O_11_	0.3	293.1236/143.0710/ 131.0708/99.0811	Flowers	[28,29]
4	5.42	625.1410	627.1832	Quercetin-3-O-gentiobioside	C_27_H_30_O_17_	−0.1	209.0293/191.0190/ 463.0866/301.0346/ 151.0031	Flowers/leaves	[30]
5	5.49	325.0932		p-Coumaroyl-O- hexoside	C_15_H_18_O_8_	1.0	163.0395/119.0498	Flowers/ leaves	[31]
6	5.80		449.1083	Cyanidin-3-O- glucoside	C_21_H_20_O_11_	−0.2	287.0549	Flowers	[32]
7	5.89	609.1460		Luteolin-diglucoside	C_27_H_30_O_16_	−0.3	447.0923/446.0551/ 285.0398/283.0244/ 151.0030	Flowers/leaves	[30]
8	6.28	695.1471		Kaempferol- malonylhexose- hexose	C_30_H_32_O_19_	0.5	651.1576/531.1151/ 489.1046/446.0859/ 285.0402	Flowers/leaves	[33]
9	6.55	651.1578		2-O-Acetylrutin	C_29_H_32_O_17_	1.7	489.1041/446.0857/ 285.0401	Flowers/ leaves	[27]
10	6.56	635.2675		Coelovirins D	C_21_H_47_O_21_	−1.5	349.1143/293.1236/ 277.1286/143.0707	leaves	[28,29]
11	6.72	473.2021		Coelovirin B	C_21_H_30_O_12_	−1.5	115.0750	Flowers/ leaves	[28,29]
12	6.91	325.0932		*p*-Coumaroyl-O- hexoside	C_15_H_18_O_8_	1.0	163.0395/119.0498	Flowers/ leaves	[31]
13	7.34	787.3220		Unknown	-	-	473.1696/285.0981	Flowers/ leaves	-
14	7.59	593.1507		Luteolin-O-rutinoside	C_27_H_30_O_19_	−0.4	285.0396	Flowers/ leaves	[27]
15	7.65	447.0929		Kaempferol-hexoside	C_21_H_20_O_11_	−0.3	284.0323/255.0295/ 227.0346	Flowers	[34]
16	7.68	457.2059		Unknown	C_21_H_30_O_11_	-	153.0550/127.0758/ 99.0809	Flowers/ leaves	-
17	7.88	887.3233		Dactylorhin A	C_40_H_55_O_22_	1.1	619.2239/439.1606/ 179.0558/153.0553	Flowers/ leaves	[35]
18	8.15	385.1436		Unknown	-	-	177.0551/145.0289/ 117.0332	leaves	-
19	8.37	771.2741		Militarine	C_34_H_46_O_17_	−1.0	457.1220/285.0979/ 153.0555	Flowers/ leaves	[35]
20	8.57	533.1344		Unknown	C_21_H_29_O_17_	2.2	390.0738/333.0760	Flowers/ leaves	-
21	8.78	1033.3541		Unknown saponin	-	-	765.2605/436.1606/ 619.2237/325.0923	Flowers	-
22	9.21	753.2615		Unknown	-	-	439.1609/153.0554	leaves	-
23	9.49	1063.3664		Unknown saponin	-	-	749.2662/569.2034/ 439.1611/153.0554	Flowers	-

## 3. Materials and Methods

### 3.1. Chemicals

Octyl octanoate (98%), alkane mix (C_6_–C_35_), formic acid, and anhydrous sodium sulfate were obtained by Sigma-Aldrich, Inc. (Milan, Italy). Diethyl ether, *n*-hexane, ethyl acetate, acetonitrile, and methanol were purchased from Merck (Darmstadt, Germany) and used without further purification. For UPLC/MS analyses, acetonitrile and formic acid LC-MS grade were provided by Romil (Cambridge, UK), and ultrapure water was produced using the Milli Q-Milli RO system, Millipore (Burlington, MA, USA).

### 3.2. Plant Material

Roots, leaves, stems, and inflorescences of *O. purpurea* were collected in April 2021 in Pianlago Ponzone (Alessandria, Italy, 44°35′21″ N 8°27′37″ E) according to the regional law and with the legal permission of the regional authorities. Plants were identified according to Chase et al. [8]. A voucher specimen of the species is deposited in the living collection of the Department of Drug Sciences (Pavia, Italy) with the accession number Op02. The plant materials were collected and immediately placed in a PVC bag and stored at +4 °C, and subsequently stored in dark conditions at −20 °C until extraction procedures.

### 3.3. Extraction of O. purpurea

#### 3.3.1. Steam Distillation

Samples of roots, stems, leaves, and flowers of *O. purpurea* (10.69 g, 58.21 g, 60.96 g, and 60.92, respectively) were spiked with octyl octanoate (35 mg) as an internal standard, and next steam distilled for 3 h. Steam distillation was performed according to De Agostini et al., 2022 [36]. Briefly, the natural matrix was placed over a stainless steel plate inside the body made of heat-resistant glass. The steam passing through the natural matrix was next cooled through a water condenser, thereby producing the essential oil and aqueous plant extract (hydrosol) simultaneously. The hydrosol was extracted with diethyl ether (3 × 100 mL), dried over anhydrous Na_2_SO_4_, concentrated under reduced pressure, and finally the solvent was completely evaporated using a gentle N_2_ stream. The obtained extract was stored at −20 °C until GC/FID and GC/MS analyses.

#### 3.3.2. Soxhlet Extraction

Samples of leaves and flowers of *O. purpurea* (26.17 g and 5.95 g, respectively) were placed in the Soxhlet apparatus and extracted sequentially with *n*-hexane, ethyl acetate, and methanol (3 × 500 mL each). The mixture was refluxed for 60 min, filtered, resuspended in fresh solvent, and refluxed for further 60 min. The fractions were collected, and the solvent removed under vacuum. The samples were successively extracted, following the procedure described above, with ethyl acetate and methanol. All dried extracts were stored at −20 °C until UHPLC-MS/MS analysis.

### 3.4. Gas Chromatographic Analysis

GC-FID analyses were carried out using an Agilent model 5980 GC (Agilent Technologies, Lexington, CA, USA), equipped with Elite-5MS (5% phenyl methyl polysiloxane) capillary column (30 m × 0.32 mm i.d.) and film 0.32 μm thick (Agilent Technologies, Lexington, CA, USA). The carrier gas was He at a flow of 1 mL/min. Aliquots of 1 μL of each essential oil after dilution (1 mg/mL) with dichloromethane were manually injected in “split” mode (30:1) with a column temperature program of 40 °C for 5 min, then increased to 260 °C at 4°C/min, and finally held at this last temperature for 10 min. The injector and detector were set at 250 and 280 °C, respectively. The flow conditions for the FID detector were 40 mL/min for hydrogen and 400 mL/min for air. The relative amount of each component was calculated based on the corresponding FID peak area without response factor correction. 

The same conditions were also used for GC-MS analyses using a GC Model 6890 N, coupled to a benchtop MS Agilent 5973 Network (Agilent Technologies, Lexington, CA, USA). The ion source temperature was set at 200 °C, while the transfer line was at 300 °C. The acquisition range was 40–500 amu in positive electron-impact ionization (EI) mode using an ionization voltage of 70 eV.

The identification of the volatile metabolites was performed by their retention indices (RI), their mass spectra, and by comparison with a NIST database mass spectral library, as well as with literature data [37,38,39]. Retention indices were calculated columns using *n-*-C_6_–C_35_ alkanes. The quantitative data were obtained from GC/FID analyses by an internal standard method and assuming an equal response factor for all detected compounds.

### 3.5. UPLC-MS Analysis

Qualitative analyses of flower and leave methanol extracts were performed using a Water ACQUITY UPLC system coupled with the high-resolution mass spectrometer (HRMS/MS) Waters Xevo G2-XS QTof (Waters Corp., Milford, MA, USA). The chromatographic separation was performed using a Biphenyl column (100 × 2.1 mm, 2.6 µm; Phenomenex, Torrance, CA, USA). The mobile phase consisted of water (A) and acetonitrile (B) both acidified to 0.1% (*v*/*v*) formic acid. The linear gradient was set at 0.0–10.0 min, 5–95% B, after each run of 5.0 min of column washing (95% B), and 5.0 min of column equilibration (5% B) before the next injection was used, and the flow rate used was 0.4 mL/min. The injected volume was set at 5.0 µL of each sample at a concentration of 0.5 mg/mL. The Xevo GS-XS QTof mass spectrometer was used in both ionization modes (positive and negative) to acquire full-scan MS and HRMS/MS analysis. The calibration of the mass spectrometer used 0.5 M sodium formate and leucine-enkephalin (200 pg/mL) as LockMass (*m*/*z* 556.2771 in positive and 554.2615 in negative ionization), infused simultaneously with the flow of column at 2 µL/min and acquired for 0.5 s every 15 s. The following experimental conditions were adopted for the electrospray (ESI) source: capillary voltage of 2.0 kV, source temperature of 150 °C, and desolvation temperature of 500 °C. High-purity nitrogen gas was used as a desolvation gas at a flow rate of 1000 L/h. MS spectra were acquired by full-range acquisition covering a mass range from 50 to 1200 *m*/*z*. The HRMS/MS acquisition was performed by data-dependent scan (DDA) experiments where the two most intense ions from the HRMS scan event were selected and subjected to collision-induced dissociation (CID) by applying a minimum signal threshold of 250, an isolation width at 2.0, and collision energy normalized to 30%. A resolving power of 30,000 both in full and in MS/MS scan modes was used. Compounds’ deconvolution was attributed using UNIFI Portal software v1.9 SR4 (Waters Corp., Milford, MA, USA) comparing MS/MS spectra with a proprietary scientific library (Traditional Medicine Library) or ChemSpider, and confirmation with the scientific literature. The MassLynx software (version 4.2, Waters Corp., Milford, MA, USA) was used for instrument control and data acquisition.

## 4. Conclusions

To conclude, the present work fills the gap related to the characterization of the phytochemical profile of *Orchis purpurea*, a herbaceous plant characterizing the Italian flora but still underexplored. The high amount of coumarin in all the organs of *O. purpurea* is of particular interest, this metabolite mainly being present in genera belonging to the Araliales, Rutales, and Asterales orders [40]. In the Orchideaceae family, simple coumarin has already been identified in a few species, mainly belonging to the genus *Dendrobium*, even if in percentages much lower with respect to the ones reported in the present work [12,16]. Taken together, the herein presented results shed a light on both the defensive strategy and the chemotaxonomy of *O. purpurea*. However, our results are only descriptive and should not push the reader to consider this organism as a possible source of coumarin, it being a plant protected by many national laws and whose harvesting is strictly regulated.

## Figures and Tables

**Table 1:** Chemical composition of essential oils from roots, stems, leaves, and flowers of *Orchis purpurea*.

Compound	CAS			Roots	Stems	Leaves	Flowers	Identification ^d^
		AI ^a^	AI ^b^	% ^c^	%	%	%	
Octane	111-65-9	800	800	0.07 ± 0.44	-	-	0.06 ± 0.04	STD, MS, RI
Furfural	98-01-1	836	831	-	-	0.09 ± 0.07	0.08 ± 0.05	MS, RI
Diacetone alcohol	123-42-2	841	840	1.11 ± 0.24	0.08 ± 0.07	-	-	MS, RI
Furfuryl alcohol	98-00-0	855	855	-	-	-	0.21 ± 0.06	MS, RI
1-Hexanol	111-27-3	871	870	0.08 ± 0.06	-	-	0.08 ± 0.06	MS, RI
Heptanal	111-71-7	902	902	-	-	-	0.05 ± 0.03	MS, RI
Unidentified	-	-	907	-	-	0.07 ± 0.06	0.22 ± 0.06	-
Benzaldehyde	100-52-7	960	958	0.07 ± 0.08	-	0.07 ± 0.06	0.10 ± 0.04	MS, RI
Phenol	108-95-2	985	985	0.07 ± 0.06	-	-	-	MS, RI
1-Decene	872-05-9	990	991	0.11 ± 0.08	-	-	-	MS, RI
Octanal	124-13-0	999	1003	0.08 ± 0.07	-	-	-	MS, RI
2,4-Heptadienal	4313-03-5	1010	1010	-	-	-	0.07 ± 0.05	MS, RI
2- Ethylhexanol	104-76-7	1031	1031	0.07 ± 0.06	-	0.05 ± 0.04	0.11 ± 0.07	MS, RI
Benzyl alcohol	100-51-6	1032	1034	0.08 ± 0.08	-	0.05 ± 0.04	-	MS, RI
Phenylacetaldehyde	122-78-1	1042	1042	0.14 ± 0.11	-	-	0.09 ± 0.05	MS, RI
Acetophenone	98-86-2	1065	1065	0.14 ± 0.11	0.13 ± 0.10	0.05 ± 0.03	-	MS, RI
*p*-Cresol	106-44-5	1076	1079	1.00 ± 0.20	11.58 ± 0.32	0.44 ± 0.11	12.68 ± 0.20	MS, RI
2-Phenyl-2-propanol	617-94-7	1089	1086	0.13 ± 0.07	0.11 ± 0.09	-	-	MS, RI
Nonanal	124-19-6	1100	1105	0.21 ± 0.14	0.13 ± 0.11	0.05 ± 0.04	0.64 ± 0.06	MS, RI
(*2E*)-2-Nonen-1-al	2463-53-8	1162	1161	-	-	-	0.09 ± 0.04	MS, RI
1-nonanol	143-08-8	1169	1173	0.13 ± 0.10	0.07 ± 0.06	-	-	MS, RI
Unidentified	-	-	1185	-	0.12 ± 0.12	0.07 ± 0.06	0.10 ± 0.06	-
1-Dodecene	112-41-4	1190	1192	0.18 ± 0.12	-	-	-	MS, RI
*p*-Methylguaiacol	93-51-6	1192	1193	-	0.08 ± 0.07	0.04 ± 0.03	0.37 ± 0.09	MS, RI
Decanal	112-31-2	1200	1206	0.17 ± 0.12	0.11 ± 0.09	0.04 ± 0.04	0.09 ± 0.05	MS, RI
*p*-vinylphenol	2628-17-3	1221	1220	0.17 ± 0.12	-	-	1.20 ± 0.06	MS, RI
2,3-Dihydro-benzofuran	496-16-2	1221	1221	-	0.06 ± 0.06	-	-	MS, RI
3-(1-Methylethyl) phenol	618-45-1	1228	1229	0.09 ± 0.09	-	-	-	MS, RI
3,5-Dimethoxy-toluene	4179-19-5	1264	1267	-	-	-	0.19 ± 0.06	MS, RI
Nonanoic acid	112-05-0	1271	1276	0.08 ± 0.06	-	-	0.67 ± 0.05	MS, RI
Unidentified	-	-	1308	-	-	-	0.41 ± 0.08	-
2-Methoxy-4-vinylphenol	7786-61-0	1315	1315	0.16 ± 0.13	0.08 ± 0.06	-	0.08 ± 0.06	MS, RI
(*2E*,*4E*)-2,4-Decadienal	25152-84-5	1317	1317	0.21 ± 0.20	-	-	0.08 ± 0.07	MS, RI
*p*-Hydroxybenzyl alcohol	623-05-2	1357	1356	0.11 ± 0.13	-	-	0.11 ± 0.06	MS, RI
Decanoic acid	334-48-5	1372	1372	-	-	-	0.05 ± 0.04	MS, RI
Unidentified	-	-	1379	0.17 ± 0.16	-	-	0.11 ± 0.06	-
3,4-dihydro-coumarin	119-84-6	1378	1384	-	0.12 ± 0.08	0.07 ± 0.03	0.08 ± 0.07	MS, RI
(*E*)-damascenone	23726-93-4	1385	1386	-	-	-	0.12 ± 0.06	MS, RI
2-Tetradecene	26952-13-6	1389	1393	0.22 ± 0.17	-	-	-	MS, RI
Tetradecane	629-59-4	1400	1400	-	-	-	0.05 ± 0.04	STD, MS, RI
Coumarin	91-64-5	1445	1455	32.30 ± 0.32	85.98 ± 0.27	98.79 ± 0.34	69.64 ± 0.22	MS, RI
2,6-Di-tert-butyl-p-benzoquinone	719-22-2	1469	1469	-	-	-	0.11 ± 0.06	MS, RI
Unidentified	-	-	1560	-	-	-	0.31 ± 0.12	-
Dodecanoic acid	143-07-7	1565	1566	0.26 ± 0.17	-	-	0.23 ± 0.12	MS, RI
1-Hexadecene	629-73-2	1590	1592	0.21 ± 0.15	-	-	0.20 ± 0.09	MS, RI
Hexadecane	544-76-3	1600	1600	0.10 ± 0.08	-	-	-	STD, MS, RI
Methyl dihydro jasmonate	24851-98-7	1656	1657	0.16 ± 0.15	-	-	-	MS, RI
Tetradecanoic acid	544-63-8	1780	1765	0.16 ± 0.14	-	-	0.24 ± 0.10	MS, RI
1-Octadecene	112-88-9	1790	1794	0.19 ± 0.20	-	-	-	MS, RI
Octadecane	593-45-3	1800	1800	0.15 ± 0.11	-	-	-	MS, RI
1-Methylethyl tetradecanoate	110-27-0	1828	1828	0.14 ± 0.10	-	-	-	MS, RI
Cyclohexadecane	295-65-8	1881	1881	-	-	-	0.46 ± 0.07	MS, RI
1-Hexadecanol	36653-82-4	1876	1887	14.03 ± 0.21	-	-	-	MS, RI
Nonadecane	629-92-5	1900	1900	0.12 ± 0.11	-	-	0.10 ± 0.05	STD, MS, RI
7,9-Di-tert-butyl-1-oxaspiro-(4,5)-deca-6,9-diene	82304-66-3	1929	1923	0.06 ± 0.06	-	-	-	MS, RI
Hexadecanoic acid	57-10-3	1972	1972	3.51 ± 0.12	-	-	2.54 ± 0.12	MS, RI
Ethyl hexadecanoate	628-97-7	1995	1995	0.09 ± 0.09	-	-	0.15 ± 0.07	MS, RI
Isopropyl palmitate	142-91-6	2026	2026	0.09 ± 0.06	-	-	-	MS, RI
(*E*)-15-heptadecenal	700381-35-7	2085	2085	43.39 ± 0.40	0.20 ± 0.11	-	0.86 ± 0.08	MS, RI
Heneicosane	629-94-7	2100	2100	-	0.12 ± 0.06	-	0.88 ± 0.08	STD, MS, RI
Unidentified	-	-	-	-	-	-	0.16 ± 0.04	-
Docosane	629-97-0	2200	2200	-	0.10 ± 0.06	-	-	STD, MS, RI
Unidentified	-	-	2271	-	0.12 ± 0.11	-	-	-
Tricosane	638-67-5	2300	2300	-	0.10 ± 0.07	-	0.73 ± 0.11	STD, MS, RI
Tetracosane	646-31-1	2400	2400	-	0.21 ± 0.11	-	-	STD, MS, RI
9-pentacosene	51865-00-0	2474	2475	-	-	-	0.07 ± 0.05	MS, RI
1-Docosanol	661-19-8	2493	2493	-	-	0.03 ± 0.02	0.27 ± 0.06	MS, RI
Pentacosane	629-99-2	2500	2500	-	0.18 ± 0.15	0.04 ± 0.03	2.53 ± 0.05	STD, MS, RI
Hexacosane	630-01-3	2600	2600	-	0.19 ± 0.15	-	0.22 ± 0.07	STD, MS, RI
Heptacosane	593-49-7	2700	2700	-	0.14 ± 0.11	0.04 ± 0.03	2.10 ± 0.07	STD, MS, RI

^a^ Kovats RI according to Adams [ibidem], ^b^ RI determined on an Elite-5 column using a homologous series of *n*-alkanes, ^c^ results are the mean of three experiments ± SD. ^d^ Method of identification: STD, standard; MS, mass spectrum in comparison with library [ibidem]; RI, retention indices in agreement with literature values.

**Table 2:** Chemical classes and their relative abundance in the essential oils from *O. purpurea*.

Class	Roots	Stems	Leaves	Flowers
Acids	4.00	-	-	3.74
Alcohols	16.12	11.91	0.61	15.13
Aldeydes	44.28	0.43	0.25	2.15
Esters (among which coumarin)	32.77 (32.30)	86.10 (86.10)	98.86 (98.86)	69.87 (69.72)
Ketones	1.11	0.08	0.05	0.52
Saturated hydrocarbons	0.44	1.04	0.08	7.13
Unsaturated hydrocarbons	0.90	-	-	0.26
Unidentified	0.31	0.37	0.14	0.91
Miscellanea	0.06	-	-	0.19
Oxygenated monoterpenes	-	-	-	0.12

**Table 3:** Yields (%) of Soxhlet extraction.

	Extractive Solvent		
	*n*-Hexane	Ethyl Acetate	Methanol
Leaves	0.33%	0.16%	3.83%
Flowers	0.95%	2.05%	12.77%

## Data Availability

The original contributions presented in the study are included in the article, further inquiries can be directed to the corresponding author.
